# Orally transmitted Chagas disease outbreaks in Latin America: a comprehensive systematic review and public health implications

**DOI:** 10.3389/fpubh.2026.1911186

**Published:** 2026-09-08

**Authors:** Y. M. Useche, B. Dinatale, O. A. Bottasso, A. R. Pérez, W. Savino

**Affiliations:** 1 Laboratory of Thymus Research, Oswaldo Cruz Institute, Oswaldo Cruz Foundation, Rio de Janeiro, Brazil; 2 National Institute of Science and Technology on Neuroimmunomodulation, Oswaldo Cruz Institute, Oswaldo Cruz Foundation, Rio de Janeiro, Brazil; 3 Institute of Clinical and Experimental Immunology of Rosario and Medical Faculty of Medical Sciences, National University of Rosario (IDICER CONICET-UNR), Rosario, Argentina; 4 Rio de Janeiro Research Network on Neuroinflammation, Oswaldo Cruz Institute, Oswaldo Cruz Foundation, Rio de Janeiro, Brazil; 5 INOVA-IOC Network on Neuroimmunomodulation, Oswaldo Cruz Institute, Oswaldo Cruz Foundation, Rio de Janeiro, Brazil

**Keywords:** açai fruit, benznidazol, cardiomyopathy, DTUs, follow-up, foodborne diseases, oral Chagas disease, *Trypanosoma cruzi*

## Abstract

**Objective:**

Food-borne Chagas disease is an emerging public health concern in South America, yet key aspects of its epidemiology and clinical impact remain poorly characterized. This systematic review aimed to systematically synthesize the available evidence on food-borne Chagas disease outbreaks in order to characterize their epidemiological, clinical, laboratory, treatment, and follow-up features, and to identify major knowledge gaps.

**Methods:**

A systematic review of food-borne Chagas disease outbreaks reported between 1965 and 2024 was conducted following PRISMA-2020 guidelines (PROSPERO-CRD42024542461). Independent reviewers screened studies and extracted data on epidemiology, food sources, diagnostics, clinical presentation, treatment, follow-up, seasonality, vectors, and parasite lineages.

**Results:**

Among 835 records identified, 111 studies met the inclusion criteria, describing 114 outbreaks in six countries, comprising 1,293 cases and 77 deaths (case fatality rate: 5.95%). Reported outbreaks increased 7.1-fold during the second half of the study period. Food sources were epidemiologically identified in only 12 outbreaks, with microbiological confirmation achieved in just one of them. In a further 82 outbreaks, the implicated food vehicle was classified as suspected based on epidemiological evidence, while no food source was identified in the remaining 20 outbreaks. Açaí was the most frequently implicated food in Brazil (32/64 outbreaks), whereas fruit-based beverages predominated elsewhere. Outbreaks clustered in Amazonian and tropical regions and occurred mainly during warm and rainy seasons, with predominance of sylvatic TcI/TcIV lineages and frequent involvement of *Rhodnius spp*. vectors. The median incubation period was 16 days. Clinical information was available for 941 patients, revealing a predominantly acute febrile syndrome (83.6%), followed by headache (37.1%), myalgia (36.4%), and facial edema (25.4%). Cardiac evaluation in 501 patients showed a substantial burden of abnormalities, including pericardial effusion (29.3%), repolarization disorders (26.3%), tachycardia (18.5%), bundle branch blocks (8.4%), and heart failure (7.2%). Follow-up data suggested persistence of cardiac alterations despite treatment, while long-term serological and molecular outcomes indicated low seronegativization and possible parasite persistence even after 10 years.

**Conclusion:**

Food-borne Chagas disease remains an important mode of *T. cruzi* transmission, particularly in Amazonian and sylvatic settings. However, substantial heterogeneity, underreporting, and inconsistent diagnostic and follow-up practices limit current understanding. Standardized outbreak investigations, long-term cohort studies, and strengthened surveillance systems are urgently needed.

**Systematic review registration:**

PROSPERO 2024 CRD42024542461. Available online at: https://www.crd.york.ac.uk/PROSPERO/view/CRD42024542461.

## Introduction

Chagas disease is a potentially life-threatening illness caused by the parasite *Trypanosoma cruzi*, found from the southern U.S. to southern Argentina and Chile. In Latin America, an estimated 70–100 million people are at risk, with 30,000 new cases yearly, 9,000 from congenital infection, and nearly 12,000 annual deaths (1). The World Health Organization (WHO) prioritizes Chagas disease among neglected tropical diseases, stressing the urgency of preventive and therapeutic advances (2).

Historically, vector-borne transmission has been the principal route of *T. cruzi* infection, whereas congenital transmission remains an important mechanism for maintaining the disease burden, particularly in areas where vector control has been successful or in non-endemic settings (1–4). However, the relative importance of transmission routes varies according to geographical region and epidemiological context.

Oral transmission of *T. cruzi* is considered an ancestral route of infection that primarily affects wild mammals but also occurs in humans (5, 6). Between 1936 and 1965, only sporadic cases of oral Chagas disease were documented in Argentina, Ecuador, and Brazil (7–10). Since 1965, however, numerous food-borne outbreaks have been reported across endemic countries (5, 8). Moreover, data from DATASUS indicate that 86.63% of acute Chagas disease cases reported in Brazil between 2018 and 2021 were likely associated with oral transmission (11), suggesting that this has become the predominant route of acute infection in several regions of the country. Consistent with this epidemiological shift, the WHO recognizes oral Chagas disease as both a re-emerging and a food-borne disease (12, 13).

Regardless of the transmission route, Chagas disease progresses in two phases: acute and chronic. The chronic phase is further subdivided into an asymptomatic or indeterminate form, and a symptomatic form that may include cardiac and/or digestive involvement (1). In vector-borne and congenital cases, the acute phase lasts 2–3 months post-infection and is evidenced by circulating trypomastigotes in blood (1). Symptoms are often absent or mild and nonspecific (fever, headache, adenosplenomegaly), making diagnosis difficult. A unilateral, painless swelling of the periorbital area (Romaña sign) is highly suggestive of vector-borne transmission in endemic areas (1). In contrast, orally transmitted acute Chagas disease tends to present severe clinical manifestations and has been associated with higher case-fatality rates in some outbreaks (8), possibly due to higher parasite inocula and distinct host–parasite interactions. In the chronic phase, parasites are undetectable in blood by direct methods but persist in tissues, as shown by molecular tools and reactivations in immunocompromised hosts (14). One to three decades after infection, ~30% of patients may develop cardiac issues, and ~10% can present megavisceral (megaesophagus and/or megacolon), neurological, or mixed complications (1, 15, 16). Benznidazole (BZN) and Nifurtimox (NFX) are the only available antiparasitic treatments for Chagas disease, showing effectiveness mainly during the acute phase, especially in children (17). Their efficacy drops in the chronic stage. Therefore, early detection and treatment and appear to be critical to reduce the disease burden (1, 15–17).

In addition, the clinical complexity of Chagas disease is further compounded by the biological diversity of its etiologic agent, *T. cruzi*. The parasite itself maintains a highly intricate life cycle involving diverse triatomine vectors and a wide range of mammalian hosts across domestic, peridomestic, and sylvatic cycles. This ecological versatility is mirrored at the genetic level, with *T. cruzi* classified into seven discrete typing units (DTUs), each associated with particular geographic regions, vector species, clinical manifestations, and responses to diagnosis and treatment (18). Such parasite heterogeneity, combined with multiple transmission routes -including the increasingly relevant oral pathway- adds layers of complexity to both the understanding and management of Chagas disease.

Currently, food-borne Chagas disease has become an epidemiological challenge, requiring better understanding of its development, latency, symptoms, lethality, treatment efficacy and clinical follow-up. It is also crucial to study its association with vectors, geography, seasonality, and food sources to improve prevention and management strategies (5, 19, 20). Therefore, this study aims to characterize oral Chagas disease outbreaks through a systematic review of cases data reported between January 1965 and January 2024 in several Latin American countries.

## Materials and methods

### Search strategy and selection criteria

We conducted a systematic review of food-borne Chagas disease outbreaks reported between January 1965 and January 2024 in accordance with the PRISMA 2020 Statement. The review protocol was registered in PROSPERO on 7 June 2024 (CRD42024542461). Although the registration was completed after the literature search, all inclusion criteria, data extraction forms, and the outbreak classification system were established a priori and remained unchanged throughout the review process. Outbreaks that occurred before December 2023 but were reported after January 2024 were not included.

An electronic search for the available literature (database inception to 05 January 2024) was performed using Pubmed, Web of Science, Embase, LILACS and Scielo databases including the followed DeCS/MeSH terms: “Chagas disease,” “*Trypanosoma cruzi* infection,” “foodborne diseases,” “outbreak,” and other terms related to oral transmission by contaminated food like: “contaminated food,” “oral infection,” “oral transmission” and several synonyms were searched in titles and abstracts. Truncation was used to ensure variant terms were retrieved. No language limits were applied. Gray literature sources were searched by UYM and PAR to identify possible relevant data about outbreaks descriptions not previously included in peer-reviewed scientific publications. These sources, included PhD thesis, dissertation databases, conference abstracts, and online local newspapers reports. We also included reported cases of acute Chagas disease from Colombian and Brazilian online government health surveillance databases. The references were exported to EndNote X7.5 and transferred to the Rayyan (https://www.rayyan.ai/) online application for the selection process. Three co-authors (UYM, PAR, and DB) independently screened the titles and abstracts of all retrieved records according to the predefined inclusion and exclusion criteria, including DeCS/MeSH terms (except those related to DTU-based outbreak determination).

Full-text articles were assessed when abstracts were inconclusive. Studies lacking diagnostic confirmation of Chagas disease, evidence of oral transmission, or extractable data were excluded. Disagreements were resolved by discussion and, when necessary, by a fourth reviewer (BOA), following the recommendations of the Cochrane Handbook. Data discrepancies were subsequently reviewed and resolved by all five co-authors through comparison with the original sources.

The review question was structured according to the Population–Exposure–Outcome framework: The Population comprised individuals involved in food-borne *T. cruzi* outbreaks; the Exposure was oral *T. cruzi* infection through contaminated food or beverages; and the Outcomes included epidemiological characteristics, clinical manifestations, laboratory findings, treatment, and follow-up outcomes.

### Data extraction

Data from the included studies were independently extracted by three authors (UYM, PAR, and DB) using an Excel form. We collected data from each outbreak, regardless of whether they involved unique or multiple outbreaks or incomplete information. Epidemiological data (date, location, country, state, city), demographic characteristics (age, sex, number of infected and deceased, latency period, and risk group), and attack rates (for exposed individuals without lab confirmation) were recorded. Diagnostic methods and clinical findings, especially cardiological alterations, were extracted. We also registered data on food contamination sources, parasite and triatomine species, and the authors' names and publication year. Authors were contacted for clarification when necessary.

### Definition of food-borne *T. cruzi* outbreak

For the purposes of this systematic review, a food-borne *T. cruzi* outbreak was defined as an event involving at least two individuals, each with at least one laboratory-confirmed positive test for *T. cruzi* infection, or by clinical confirmation in an epidemiologically-linked case. Additionally, food-borne *T. cruzi* outbreak required the sharing of epidemiological or Chagas disease's clinical characteristics and/or evidence of common spatial and temporal exposure to the parasite. This definition aligns with standard criteria for foodborne disease outbreaks established by the World Health Organization (21), and the US Centers for Disease Control and Prevention (22), and is consistent with definitions used in the literature for orally transmitted *T. cruzi* outbreaks (23, 24). Reports involving only one person were excluded due to difficulty in distinguishing transmission routes, except for clearly identified index cases or outbreak-related cases.

### Assessment of study quality

Given the scarcity of analytical studies on oral outbreaks of Chagas disease and the heterogeneity of the available evidence on oral *T. cruzi* transmission, mostly consisting of case reports and outbreak descriptions, we assessed study quality using the JBI Critical Appraisal Tool for case reports (25, 26), adapted to assess other descriptive reports and gray-literature sources (https://jbi.global/critical-appraisal-tools). Each checklist item was rated as Yes, No, Unclear, or Not applicable, following JBI methodological guidance. The appraisal was used to identify methodological strengths and limitations relevant to each report.

### Consolidation of outbreak evidence

When multiple reports referred to the same outbreak, they were grouped under a single Outbreak ID and data were consolidated to avoid double counting and improve completeness. Outbreaks were classified as: (A) Well-documented, based on clinical and epidemiological evidence of oral Chagas disease with reliable identification of contaminated food, with additional support from one or more of the following data when available: detection of *T. cruzi* in food and/or local triatomines; DTU characterization or DTU concordance between patients and food or vectors, and exclusion of alternative transmission routes, thereby reinforcing oral transmission; (B) Partially-documented: clinical evidence with temporal and spatial clustering of acute cases and suspected or not identified food source, with also optional evidence of local vector detection and/or DTU concordance between patients and vectors; (C) Scarcely-documented, comprising clustered confirmed oral Chagas infections with major gaps in clinical or epidemiological information.

This framework allowed standardized appraisal of evidence robustness and limitations across the range of descriptive evidence types included in this systematic review.

### Statistical analysis and graph

Descriptive statistics are presented as frequencies for categorical variables, means and standard deviations for normally distributed data, or median with range or quartiles for other continuous variables. Categorical variables were compared using the chi-square test or Fisher's exact test. The geographical distribution maps were created using Scimago Graphica (https://www.graphica.app/), incorporating geolocation data obtained from bibliographic sources or Google Maps.

### Role of the funding source:

The funder of the study had no role in the study design, data collection, analysis, and interpretation or writing the report.

## Results

### Studies included

**Figure 1:**
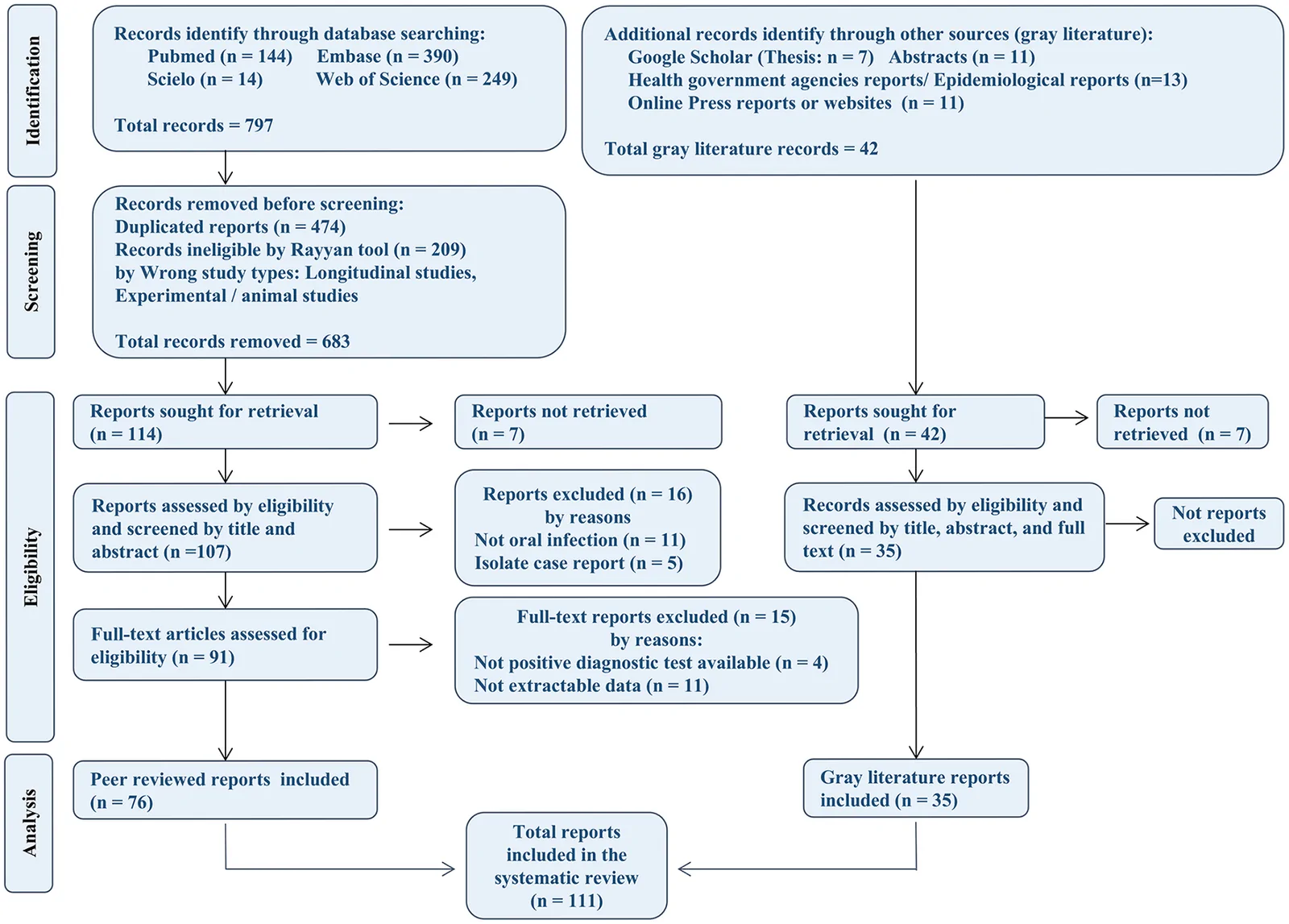
Flowchart of the study selection process for the systematic review.

In accordance with PRISMA guidelines, 797 records from bibliographic databases and 42 from gray literature were identified (total = 835). After screening, were excluded duplicates or ineligible reports, including *in vitro* and/or animal studies. Full-text assessment excluded studies without a clear definition of oral infection, isolated case reports, or extractable data, while molecular typing–only studies were included. Gray literature sources (theses, conference or congress abstracts, official epidemiological reports and press reports for seasonality analysis) were considered. The study selection process is shown in the PRISMA flow chart (Figure 1).

Ultimately, 76 peer-reviewed articles and 35 gray literature texts were included yielding 111 source reports of 114 food-borne Chagas disease outbreaks reported between 1965 and January 2024. Overall, 1,293 infected individuals were reported across the included outbreaks. Because not all studies provided complete clinical, laboratory, or follow-up information, the denominator for each analysis is indicated throughout the Results. Source reports are detailed in Table 1.

**Table 1:** Summary of food-borne Chagas disease outbreaks reported between January 1965 and January 2024 (^*^).

Country	#ID	Outbreak year	Implicated food	Food source evidence	Cases	Sources reports
Bolivia Brazil	1	2010	Majo juice	Identified	14	(44)
	2	NA	Açaí	Suspected	3	(45)
	3	1965	Meal	Not identified	17	(46, 47)
	4	1975	Not reported	Not identified	20	(48)
	5	1983	Not reported	Suspected	3	(48)
	6	1984	Açaí	Suspected	2	(49)
	7	1984	Açaí	Suspected	6	(49)
	8	1985	Not reported	Not identified	3	(50)
	9	1986	Raw cane juice	Identified	26	(51)
	10	1988	Not reported	Not identified	5	(48)
	11	1989	Not reported	Not identified	3	(48)
	12	1991	Not reported	Not identified	4	(48)
	13	1992	Not reported	Not identified	2	(50)
	14	1992	Not reported	Not identified	5	(48)
	15	1994	Urucuri	Suspected	3	(48)
	16	1996	Not reported	Not identified	2	(48, 52)
	17	1996	Not reported	Not identified	4	(48, 52)
	18	1996	Açaí	Suspected	17	(48, 52, 53)
	19	1998 (?)	Açaí	Suspected	4	(48)
	20	1998 (?)	Açaí	Suspected	4	(48)
	21	1998	Meal	Suspected	13	(54, 55)
	22	1998	Açaí	Suspected	2	(48)
	23	1998	Fruits	Suspected	16	(48)
	24	1999	Bacaba	Suspected	13	(56)
	25	1999	Not reported	Not identified	3	(48, 57)
	26	1999	Açaí	Suspected	7	(58)
	27	2000	Beverages	Suspected	11	(48, 59)
	28	2000	Açaí	Suspected	6	(48)
	29	2000	Açaí	Suspected	2	(48)
	30	2000	Açaí	Suspected	6	(48)
	31	2001	Açaí	Suspected	10	(48, 60)
	32	2001	Açaí	Suspected	6	(48)
	33	2002	Not reported	Not identified	10	(48)
	34	2002	Açaí	Suspected	10	(48)
	35	2003	Açaí	Suspected	9	(61)
	36	2004	Açaí	Suspected	9	(50, 61–64)
	37	2004	Açaí	Suspected	14	(48)
	38	2004	Açaí	Suspected	27	(6, 65, 66)
	39	2005	Sugar cane juice	Identified	31	(67–71)
	40	2006	Meal and beverages	Suspected	7	(72, 73)
	41	2006	Sugarcane juice	Identified	6	(73)
	42	2006	Bacaba or açaí juice	Suspected	21	(48, 74)
	43	2006	Soup and herbs	Suspected	8	(75)
	44	2006	Açaí	Suspected	12	(61, 76)
	45	2006	Açaí	Suspected	4	(77)
	46	2006	Açaí	Suspected	12	(61)
	47	2007	Açaí	Suspected	25	(62, 63, 74, 78, 79)
	48	2007	Açaí and meal	Suspected	13	(80)
	49	2007	Açaí and meal	Suspected	12	(80)
	50	2007	Meal	Suspected	3	(81)
	51	2009	Açaí	Identified	17	(82)
	52	2011	Açai	Suspected	15	(62, 63)
	53	2012	Not reported	Not identified	6	(83)
	54	2015	Sugar cane juice	Suspected	18	(84, 85)
	55	2015	Açai	Suspected	19	(62, 63)
	56	2016	Bacaba wine	Suspected	9	(35, 86–88)
	57	2016	Açaí	Suspected	5	(89)
	58	2017	Açaí	Identified	20	(62, 63, 90)
	59	2017	Patauá juice	Suspected	7	(35)
	60	2018	Bacaba juice	Identified	39	(91)
	61	2019	Patauá juice	Suspected	16	(63, 92)
	62	2019	Açaí	Suspected	15	(63, 93–95)
	63	2019	Not reported	Not identified	40	(96–98)
	64	2021	Açaí	Identified	5	(63, 99)
	65	2022	Açaí	Suspected	15	(63)
Colombia	66	1992	Not reported	Not identified	14	(36, 100–102)
	67	1999	Palm wine	Suspected	15	(36, 67, 101, 103)
	68	2003	Contaminated water	Suspected	3	(36)
	69	2008	Tangerines and orange juice	Identified	10	(36, 37, 101, 104–106)
	70	2009	Not reported	Not identified	5	(101)
	71	2009	Meal or water	Suspected	11	(101, 102, 107)
	72	2009	Mandarine/orange juice	Suspected	5	(37, 101, 106)
	73	2009	Not reported	Not identified	5	(37, 101, 106)
	74	2010	Not reported	Not identified	11	(36, 101)
	75	2010	Armadillos/opossums meat	Suspected	2	(37)
	76	2010	Not reported	Not identified	3	(36, 37, 101)
	77	2010	Shared meal	Suspected	5	(37, 101)
	78	2014	Pineapple juice	Suspected	4	(108)
	79	2014	Meal or water	Suspected	42	(108–110)
	80	2015	Contaminated food	Suspected	8	(111)
	81	2017	Not reported	Not identified	11	(112)
	82	2017	Not reported	Not identified	2	(112)
	83	2017	Not reported	Not identified	4	(112, 113)
	84	2019	Meal	Suspected	4	(102, 114)
	85	2019	Meal	Suspected	3	(102, 114)
	86	2019	Contaminated water	Suspected	16	(102, 115)
	87	2019	Contaminated water	Suspected	22	(102, 114, 116)
	88	2019	Contaminated water	Suspected	4	(102, 114)
	89	2019	Armadillo blood	Identified	2	(102, 114)
	90	2019	Sugar cane juice, hunted bushmeat	Suspected	2	(117)
	91	2020	Not reported	Not identified	4	(102, 118, 119)
	92	2021	Not reported	Not identified	9	(120, 121)
	93	2021	Meal or water	Suspected	10	(122)
	94	2022	Not reported	Not identified	3	(102, 123)
	95	2023	Wild animal meat	Suspected	10	(102, 124)
French Guiana	96	2005	Bacaba fruit juice	Suspected	8	(125)
Perú	97	2010	Masato juice	Suspected	2	(126)
Venezuela	98	2005	Not reported	Not identified	7	(127)
	99	2007	Guava juice	Identified	109	(27, 38, 128–134)
	100	2007	Not reported	Not identified	9	(127, 135)
	101	2007	Not reported	Not identified	2	(135)
	102	2008	Not reported	Not identified	3	(38, 127)
	103	2009	Guava juice	Identified	88	(38, 127, 136–140)
	104	2010	Not reported	Not identified	6	(38, 127, 141, 142)
	105	2010	Passion fruit juice	Suspected	22	(38, 127, 138, 142)
	106	2012	Banana and papaya fruits	Suspected	5	(127, 143)
	107	2012	Not reported	Not identified	4	(127)
	108	2013	Mango juice	Suspected	9	(48, 127, 144)
	109	2014	Pomarosa juice	Suspected	3	(127, 142, 144)
	110	2014	Not reported	Not identified	5	(127)
	111	2015	Yuca homemade tea and other beverages	Suspected	2	(145)
	112	2015	Meal and Guava	Suspected	9	(146)
	113	2015	Contaminated water	Suspected	3	(147)
	114	2018	Home-made fruit ice cream	Suspected	27	(148)

(^*^), Outbreaks occurred before December 2023 but reported after January 2024 were not included. #ID, Outbreak Identfication number. (♦), Suspected outbreak year. Açaí, Euterpe oleracea or E. precatoria; Sugarcane, Saccharum officinarum; Guava, Psidium guajava; Majo fruit, Oenocarpus bataua; Bacabá, Oenocarpus bacaba; Urucuri, Attalea phalerata.

### Quality and completeness of evidence in food-borne Chagas disease outbreaks

The JBI-based Critical Appraisal Tool allows identifying methodological strengths and limitations relevant to each report, revealing substantial heterogeneity in reporting quality across outbreaks, with frequent limitations related to incomplete clinical descriptions, lack of laboratory confirmation of food contamination, and limited long-term follow-up. However, most reports fulfilled criteria related to case description and diagnostic confirmation, whereas information on food source investigation and follow-up was frequently incomplete (Supplementary Table 1). Lastly, consolidation evidence of food-borne outbreaks according to quality and quantity of extractable data from diverse reports showed that only 10 outbreaks (10/114, 8.8%) were reported with well detailed data. Among them, only one study had an unequivocal microbiological detection of the parasite in the contaminated food. Additionally, 87 outbreaks (87/114, 76.3%) were described partially, while the rest had a scarce (17/114, 14.9%) description (Supplementary Table 2).

### Characteristics of food-borne Chagas disease outbreaks

**Figure 2:**
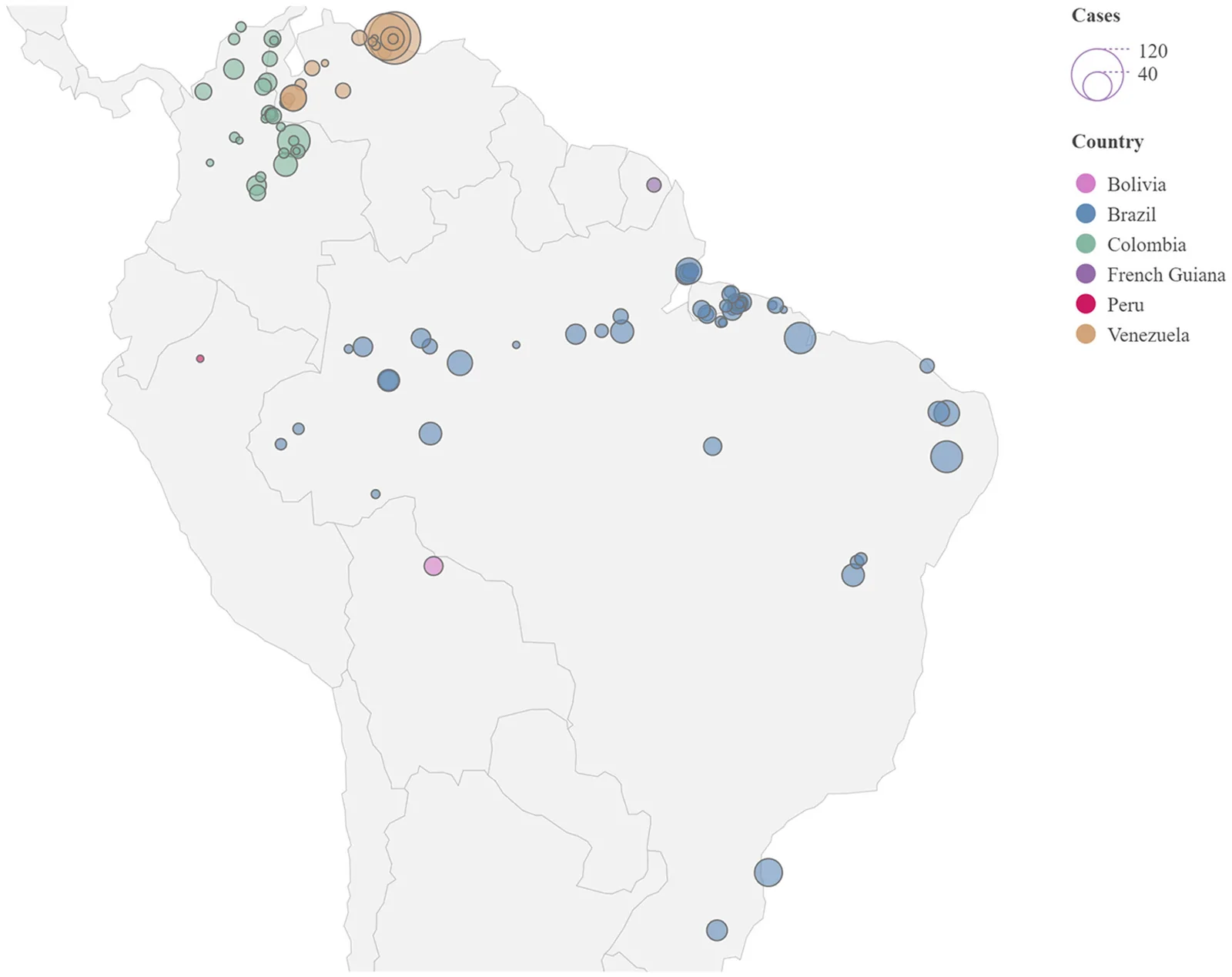
Geolocation of food-borne Chagas disease outbreaks reported between January 1965 and January 2024. Each point represents a documented outbreak (*n* = 114), with circle size proportional to the number of associated cases. Overlapping points indicate multiple outbreaks reported at the same location. Insets show magnified views of selected regions (A1 → A2, B1 → B2).

Detailed demographic and epidemiological information for each outbreak is provided in Supplementary Table 3. Systematic compilation showed that most outbreaks occurred in Brazil, Colombia, and Venezuela, with Venezuela accounting for the two largest outbreaks, each involving 88 and 109 cases. In contrast, only a single food-borne outbreak was identified in each of Bolivia, Peru, and French Guiana, and none were reported in Argentina during the study period, although sporadic cases had been documented there prior 1965. Fourteen of the reported outbreaks occurred between 1965 and 1995; however, from 1996 to 2023, this number increased to 100, representing an almost 7.1-fold increase over a comparable time span (27 years) (Table 1; Figure 2).

Deaths were reported in 37 outbreaks (37/114, 32.5%), with a total of 77 fatalities recorded during the 1965–2023 period, corresponding to an overall case fatality rate (CFR) of 5.95% (77/1,293) (Table 2).

**Table 2:** Case numbers and case fatality rate of food-borne Chagas disease outbreaks occurred by country in the evaluated period.

Country	Outbreaks (*n*)	Cases (*n*)	Total deaths (*n*)	F/M/(N.a.) (*n*)	CFR(^*^) (%)
Bolivia	1	14	N.a.	N.a/N.a./(14)	N.a.
Brazil	64	707	29	142/156/(409)	4.10
Colombia	30	249	23	46/138/(65)	9.2
French Guiana	1	8	N.a.	4/4/(0)	N.a.
Peru	1	2	0	0/2/(0)	0
Venezuela	17	313	25	134/128/(51)	7.98
Total	114	1,293	77	326/428/(539)	5.95

n, case numbers; F, female; M, male; N.a., not available. CFR^(*)^, Case fatality rate was calculated as the total reported deaths by oral Chagas disease infection/total number of diagnosed cases of oral ChD infection.

Reliable extractable data on sex were available only for 754 infected patients (754/1293, 58.3%). Females accounted for 43.2% of them (*n* = 326), while males represented 56.8% (*n* = 428). Among cases with available sex information, 27 deaths were reported in men and 15 in women, with no statistically significant difference (Fisher's exact test). Nine pregnant women orally-infected were reported, 5 of them experiencing abortions, representing a 55.5% fetal loss (Supplementary Table 3).

Age data were available in only 51 of the 114 outbreaks (44.7%). Based on these data, the mean age of infected individuals was 24.9 ± 10.6 years (SD), with a range of 1 to 80 years. Among the 37 outbreaks that reported deaths, only 16 provided age information for fatal cases; in these, the mean age was 25.2 ± 10.3 years (SD), ranging from 1 to 74 years (Supplementary Table 3).

Information on the size of the estimated exposed population was available for only 60 outbreaks (59/114, 51.7%), and underestimation of exposure in some events cannot be ruled out. Based on this data, the global attack rate was 48.1% (1,293 infected/2,688 exposed individuals). In 86 outbreaks (86/114; 75.4%), the reports described the characteristics of exposed populations, with relatives involved in more than half of these outbreaks (64/114; 56.1%), coworkers (13/114, 11.4%), neighbors or non-relatives (10/114, 8.7%), groups that consumed commercial food (8/114, 7.0%), and members of closed communities (such as schools, older adults care facilities or congregations, 7/114, 6.1%). In 16 outbreaks, the affected groups included more than one category (i.e., relatives and non-relatives) (Supplementary Table 3).

### Food sources associated with oral Chagas disease outbreak

Food sources were mentioned in 82 of the 114 outbreaks (72.0%), but identified in only 12 of them. Among these, 11 outbreaks (13.6%) had epidemiological confirmation of a contaminated food source, and one additional outbreak had microbiological confirmation (açaí, outbreak #58, Supplementary Table 3). Overall, açaí, sugarcane and fruit juice were the most frequently confirmed vehicles. However, distinct geographic patterns in food-associated transmission emerged. In Brazil, outbreaks were predominantly associated with açaí consumption (32/64; 50.0%), whereas in Colombia suspected sources more commonly included fruit juices, bushmeat, and beverages or water probably contaminated with vector feces. Venezuelan outbreaks were mainly linked to homemade fruit juices, including the two largest outbreaks reported. Other countries reported only isolated events involving locally consumed beverages or foods. Additionally, five outbreaks were suspected to be associated with water sources contaminated by feces or urine from infected vectors, or by scent-gland secretions of infected opossums.

### Geolocalization and seasonality of food-borne Chagas disease outbreaks

**Figure 3:**
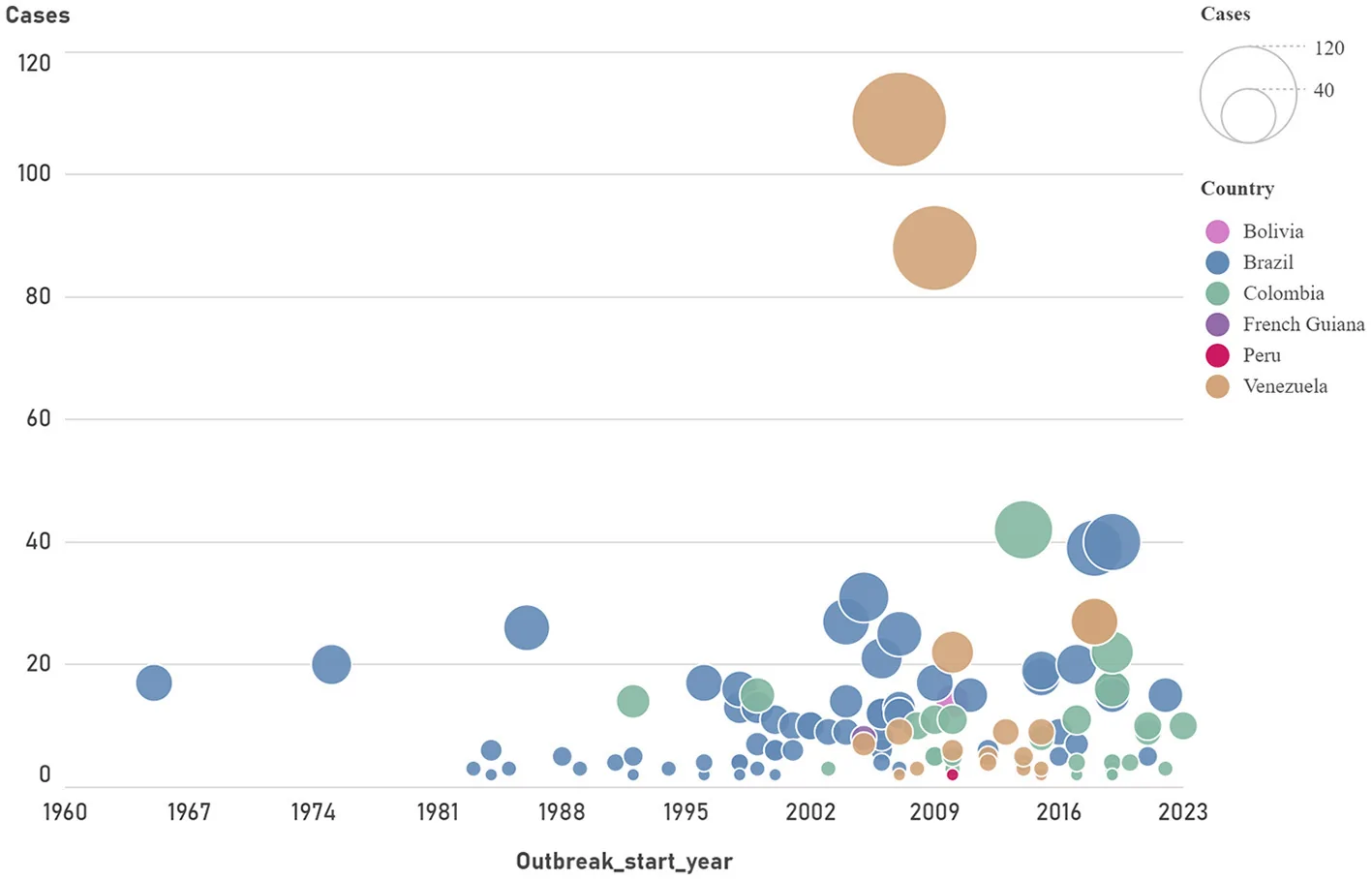
Temporal trends in reported food-borne Chagas disease outbreaks by country. Annual number of orally transmitted Chagas disease outbreaks and associated cases per outbreak in Latin American countries, January 1965-January 2024. Although the first outbreak was officially reported in 1998, it retrospectively occurred in 1965. The relative size of each outbreak, in terms of reported cases, is represented by the diameter of the circles.

As depicted in the georeferenced map (Figure 3), food-borne Chagas disease outbreaks were predominantly concentrated in sylvatic or tropical regions -mainly within the Amazon basin- as well as in subtropical areas associated with agricultural activities. Latitude and longitude data for each city or municipality in which an outbreak occurred are provided in Supplementary Table 3.

**Figure 4:**
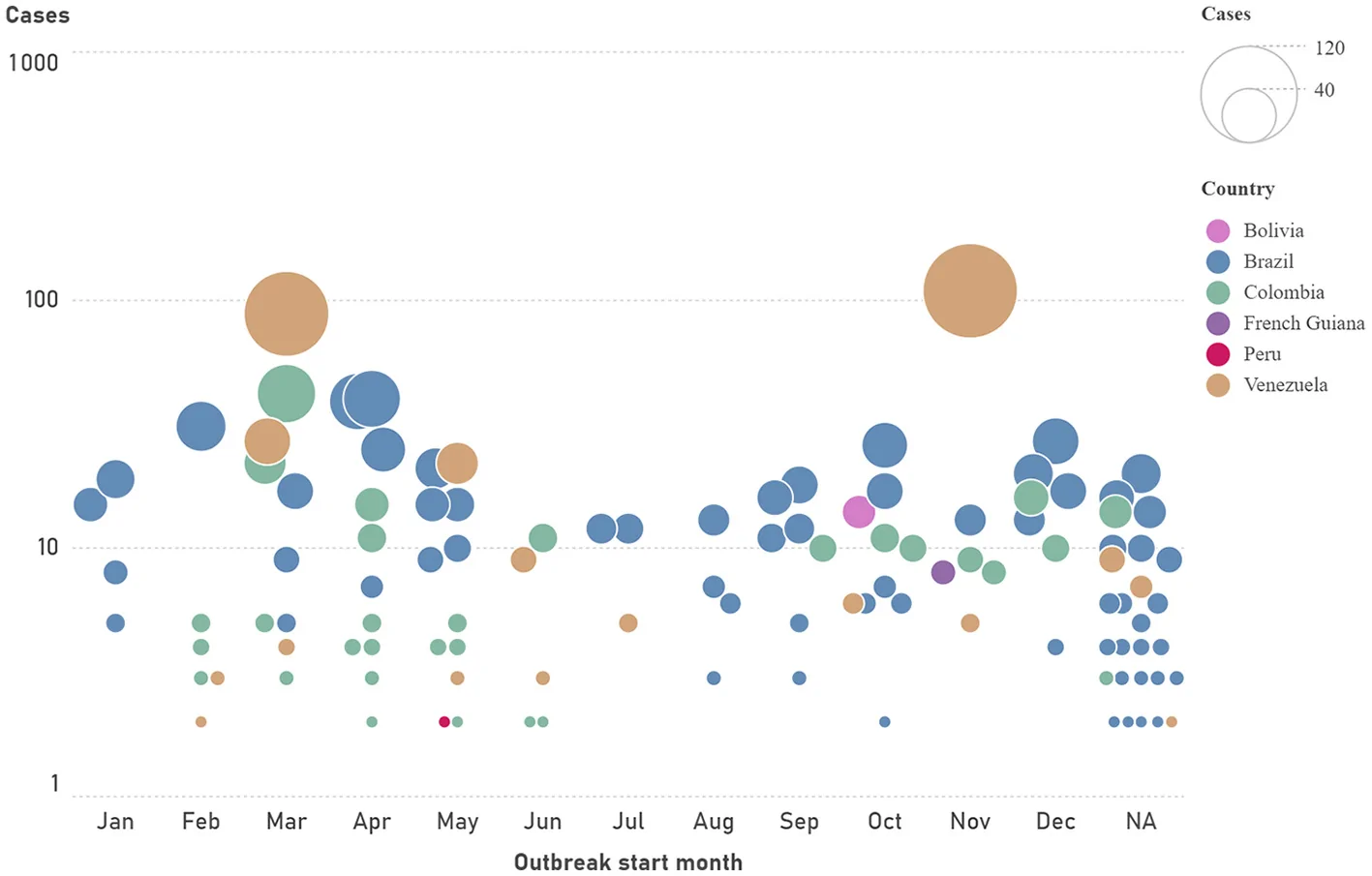
Seasonal patterns of orally transmitted Chagas disease outbreaks. The month corresponds to the reported onset of each outbreak reported between January 1965 and January 2024. Associated cases per outbreak occurred are also shown.

Seasonality data were available for 85 of 114 outbreaks (74.5%) and compiled data revealed a marked seasonal pattern, with outbreaks occurring mainly during the warmest and wettest months (Figure 4; Supplementary Table 3). The highest concentration was observed during the period from March to May (33 outbreaks, 489 cases), followed by October to December (23 outbreaks, 364 cases). Only 3 outbreaks were reported in July (29 cases). Outbreaks often coincided with tropical fruit and crop harvesting periods, suggesting a link with agricultural practices. In Brazil, açaí consumption is associated with peaks in oral transmission, with a decline from January to June, a pattern also reported for sugarcane-related outbreak in Santa Catarina.

### Molecular characterization of *Trypanosoma cruzi* DTUs in food-borne Chagas disease outbreaks

*T. cruzi* is classified into seven discrete typing units (DTUs: TcI–TcVI and Tcbat), with TcI, TcIII, and TcIV predominantly associated with sylvatic cycles, while TcII, TcV, and TcVI are mainly found in domestic transmission cycles (18). DTUs identified in each human sample, food or vector are detailed in Supplementary Table 3.

**Figure 5:**
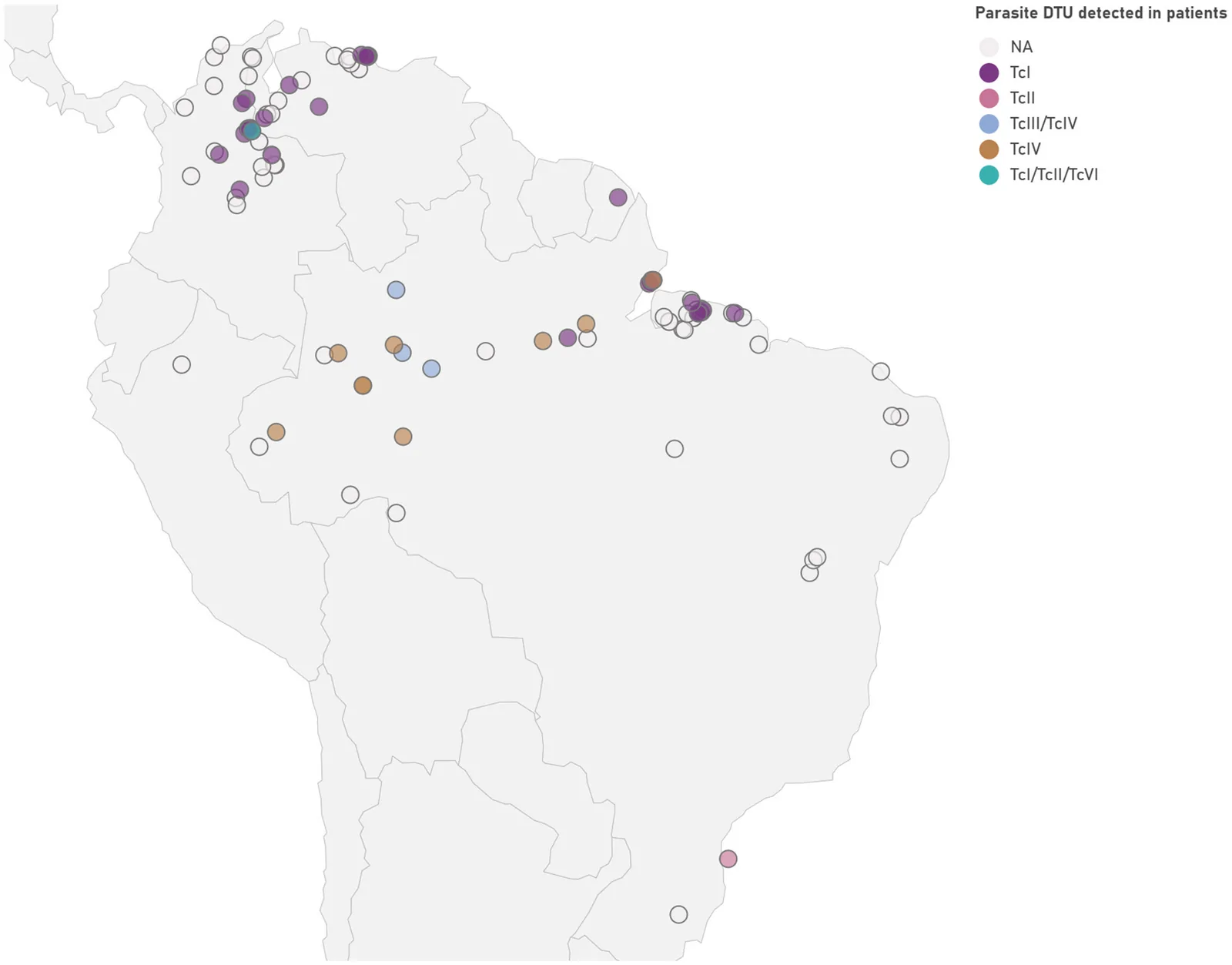
Geographic distribution of DTUs associated with food-borne Chagas disease outbreaks. Each point represents the geographic distribution of DTUs identified in human samples according to outbreak location.

In 42 outbreaks (36.8%), parasite lineages were determined from one or more patients per outbreak, predominantly involving sylvatic lineages (41/42), indicating that not all patients within these outbreaks underwent lineage typing. Moreover, in a limited number of outbreaks, molecular characterization revealed the presence of multiple *T. cruzi* lineages within the same outbreak. PCR analysis of human samples identified the following DTUs: TcI (28 outbreaks), TcII (1 outbreak), TcIII/IV (formerly Z3, 3 outbreaks), TcIV (9 outbreaks), and mixed TcI/TcII/TcVI (1 outbreak). Figure 5 illustrates the geographic distribution of the DTUs identified in human samples according to outbreak location. No TcV or TcVI lineages were detected

In only one specific outbreak (outbreak #58), DTU detection confirmed the TcIV lineage in the contaminated food.

Regarding vector-derived samples, TcI was identified in triatomines collected at the sites of 17 outbreaks: as the sole DTU in 15 outbreaks and as part of mixed infections (TcI/TcII or TcI/TcII/TcIII) in the remaining two outbreaks.

### Species of vectors suspected of being involved in food-borne Chagas disease outbreaks

The most common triatomine genera in food-borne Chagas disease outbreaks were *Rhodnius spp*. (33/114; 28.9%), followed by *Panstrongylus spp*. (22/114; 19.3%) and *Triatoma spp*. (11/114; 9.6%). *R. pictipe*s and *R. robustus* predominated in Brazil, whereas *P. geniculatus* was most prevalent in Colombia and Venezuela (Table 3). Species were not reported for outbreaks in Bolivia, French Guiana, or Peru. Details of the vectors identified or suspected to be involved in each outbreak are provided in Supplementary Table 3.

**Table 3:** Species of vectors suspected of being involved in food-borne Chagas disease outbreaks by country.

Brazil [vector/(*n* outbreaks)]	Colombia [vector/(*n* outbreaks)]	Venezuela [vector/(*n* outbreaks)]
*Rhodnius pictipes (10)*	*Panstrongylus geniculatus (8)*	*Panstrongylus geniculatus (9)*
*Rhodnius robustus (9)*	*Rhodnius pallescens (5)*	*Rhodnius prolixus (2)*
*Panstrongylus geniculatus (3)*	*Rhodnius prolixus (3)*	*Triatoma maculata (1)*
*Triatoma brasiliensis (3)*	*Eratyrus mucronatus (1)*	*Rhodnius pallescens (1)*
*Triatoma sordida (3)*	*Rhodnius pictipes (2)*	
*Panstrongylus lutzi (1)*		
*Panstrongylus megistus (1)*		
*Triatoma infestans (2)*		
*Rhodnius brethesi (1)*		
*Triatoma tibiamaculata (1)*		
*Triatoma pseudomaculata (1)*		

Data is presented as a summary of all reported species, ordered by the number of observed occurrences in each country.

Among the 17 outbreaks with identified vector species and parasite DTUs, *Rhodnius spp. and Panstrongylus geniculatus* showed a strong association with TcI isolates, whereas *Triatoma* species exhibited mixed infections (Supplementary Table 3).

### Laboratory confirmation of oral Chagas disease outbreaks

Laboratory assays were essential to confirm *T. cruzi* as the etiological agent in food-borne outbreaks, although information on the diagnostic methods used was available for only 89 outbreaks (78.1%) involving a total of 1,064/1,293 infected individuals examined using one or more of the diagnostic tests. Detailed information regarding the diagnostic methods used in each outbreak and the number of positive cases, is provided in Supplementary Table 4.

Overall, conventional parasitological and serological methods showed consistently high positivity rates, generally exceeding 85% (Table 4). Among parasitological techniques, culture yielded the highest positivity (93.2%), followed by xenodiagnosis (88.3%) and direct microscopic examination (86.0%). Likewise, serological assays demonstrated high diagnostic performance, with IFI showing the highest positivity (97.8%), followed by ELISA (95.2%) and IHA (88.3%). PCR/qPCR was reported in only a subset of outbreaks but was performed in more than 300 individuals, with an overall positivity of 86.7%, supporting its usefulness as a confirmatory diagnostic tool during acute food-borne outbreaks.

**Table 4:** Laboratory findings across diagnostic methods used in oral Chagas outbreaks.

Diagnostic category	Diagnostic technique	Examined individuals (*n*)	Positive tests (*n*)	Positivity rate (%)
Parasitologic	Direct examination	570	490	86.0
	Xenodiagnosis	223	197	88.3
	Culture	281	262	93.2
Serologic	IHA	497	439	88.3
	ELISA (IgM and/or IgG)	396	377	95.2
	IFI (IgM and/or IgG)	418	409	97.8
Molecular	PCR/qPCR	331	287	86.7
Other (^*^)		348	143	40.0

Diagnostic methods used during reported food-borne Chagas disease outbreaks and their corresponding positivity rates. Positivity rate (%) was calculated as the number of positive results divided by the number of individuals examined × 100. Data are based on a total of 1,064 individuals examined using one or more of the diagnostic tests listed. Because individual patients could undergo more than one diagnostic test, the total numbers of examined individuals and positive tests exceed the total number of infected individuals. Other tests included biopsy, necropsy, laboratory animal inoculation, TESA blot, immunoprecipitation, salivary antibody detection, and other less frequently reported methods. (^*^) For other diagnostic methods, the number of examined individuals was not consistently reported across studies. Therefore, positivity rates were calculated using the total number of patients in each outbreak as the denominator rather than the number of individuals tested.

### Clinical features of patients with acute food-borne Chagas disease

Complete epidemiological curves were available or reconstructed for only 19 outbreaks (19/114, 16.6%), showing symptom onset 2 to 31 days after exposure, with a median incubation period of 16 days (Supplementary Table 3).

**Figure 6:**
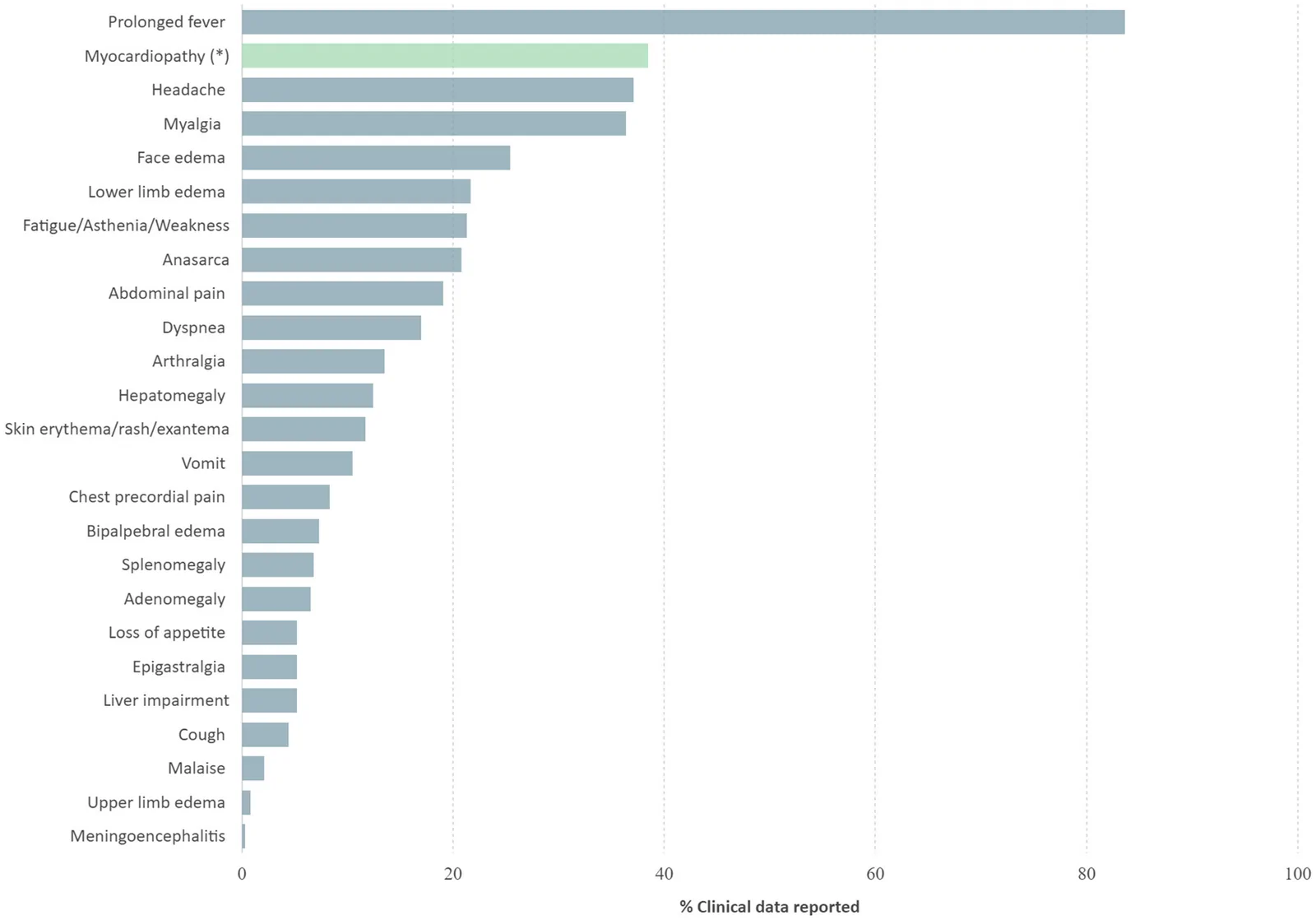
Proportion of clinical symptoms and findings in patients with acute oral Chagas disease. Gray bars represent the percentage of patients presenting each symptom or sign out of the total 769 reported individuals. The proportion of myocardiopathy (*, green bar) was calculated based on the 501 patients who underwent cardiologic evaluation.

Clinical assessments were reported for 941/1,293 infected individuals (72.8%), although detailed information was available for only 769/1,293 cases (59.5%). Among these 769 cases, prolonged fever was the most frequent manifestation (643/769; 83.6%), followed by headache (285/769; 37.1%), myalgia (280/769; 36.4%), and facial edema (195/769; 25.4%). A wide range of less frequent manifestations were also reported, including two cases of meningoencephalitis (Figure 6; Supplementary Table 5).

Cardiological evaluations were described in a minor group of patients (*n* = 501/1,293, Table 5; Supplementary Table 6), representing 38.7% of all infected patients and corresponding to 54 outbreaks. Cardiomyopathy was detected in 193 individuals (Figure 6; Table 5). ECG, Holter and echocardiographic evaluations showed that the most frequent findings were pericardial effusion (29.3%), repolarization disorders (26.3%), tachycardia (18.5%), and bundle branch blocks (8.4%), along with heart failure (7.2%) (Table 5). In addition, cardiac involvement was documented in 26 fatal cases, including one acute myocardial infarction and nine necropsy-confirmed myocarditis cases. No digestive megasyndromes were reported, however, it was not explicitly stated whether these manifestations were systematically evaluated.

**Table 5:** Cardiac abnormalities reported among patients evaluated during acute food-borne Chagas disease outbreaks determined by ECG and echocardiogram.

Cardiac evaluation and findings	Reported cases (*n*)	%
**Total number of cardiologically evaluated patients**	**501**	**100.0**
**Total patients with myocardiopathy**	**193**	**38.5**
**Repolarization disorders**	**132**	**26.3**
-Without description	57	11.3
-Other Repolarization Disorders	37	7.4
-Disturbances in Ventricular Repolarization	27	5.4
-Disturbances in Auricular Repolarization	11	2.2
**Pericardial effusion**	**147**	**29**.**3**
**Tachycardia**	**93**	**18**.**5**
-Without description	42	8.4
-Sinus tachycardia	34	6.8
-Atrial tachycardia	14	2.8
-Ventricular tachycardia	3	0.6
**Branch Blocks**	**42**	**8**.**4**
-AV block	21	4.2
-Complete right bundle branch block (cRBBB)	16	3.2
-Anterosuperior hemiblock (HB)	6	1.2
-Right bundle branch hemiblock (RBBHB)	3	0.6
-Complete left bundle branch block (LBBB)	7	1.4
**Heart failure**	**36**	**7**.**2**
**Pericarditis**	**18**	**3**.**6**
**Mildly reduced or reduced Ejection Fraction (< 49%)**	**18**	**3**.**6**
**Mitral Regurgitation**	**14**	**2**.**8**
**Atrial Fibrillation** (AF)	**12**	**2**.**4**
**Left Ventricular hypertrophy**	**31**	**6**.**2**
**QRS low voltage** (LV)	**11**	**2**.**2**
**Extrasystoles**	**9**	**1**.**8**
**Aneurysmal dilations**	**3**	**0**.**6**
**Sinus Bradycardia** (SB)	**5**	**1**.**0**
**Syncope**	**1**	**0**.**2**

Data are presented as the number and percentage of cardiac abnormalities among the total number of cardiologically evaluated patients (*n* = 501). Abnormalities were counted as reported events. Since individual reports frequently described more than one electrocardiographic abnormality in the same patient, subcategories are not mutually exclusive and therefore may not sum to the total number of affected patients. Moreover, in many reports, it was not possible to determine whether multiple abnormalities occurred in the same patient, precluding patient-level analysis.

### Cardiological follow-up of orally-infected patients

Cardiological follow-up was reported for only 15 outbreaks (15/114, 13.2%), including 178/1,293 BZL/NFX treated patients (13.8% of all infected individuals). Follow-up assessments were inconsistently reported and varied widely in timing, ranging from evaluations conducted within months after completion of antiparasitic treatment to assessments performed up to 10 years, with some patients undergoing repeated evaluations over time (Table 6).

**Table 6:** Cardiological follow-up findings in patients with orally acquired Chagas disease after antiparasitic treatment.

Follow-up period (after treatment completion)	Patients evaluated by ECG (*n*)	ECG alterations (*n*, %)	Patients evaluated by Echo (*n*)	Echo alterations (*n*, %)
0–6 months	114	42 (36.8)	118	8 (6.7)
6–12 months	114	33 (28.9)	23	6 (26.1)
13–48 months	117	18 (15.4)	34	2 (5.9)
8–10 years	71	7 (9.8)	14	6 (42.8)

Data summarizes the number of patients evaluated by electrocardiography (ECG) and echocardiography (Echo), together with the proportion presenting cardiac abnormalities at different follow-up intervals after completion of benznidazole (BZL) or nifurtimox (NFX) treatment. Because follow-up duration, evaluation schedules, and the number of patients assessed varied across outbreaks, the data represent pooled descriptive findings and should not be interpreted as longitudinal changes within the same patient cohort.

As seen in Table 6, during the post-treatment follow-up, the proportion of patients with ECG abnormalities showed a progressive decline over time, decreasing from 36.8% (0–6 months) to 9.8% (8–10 years). In contrast, the prevalence of echocardiographic alterations did not follow a consistent downward trend, exhibiting marked fluctuations across the different follow-up intervals (6.7%, 26.1%, 5.9%, and 42.8%, respectively). Notably, the number of patients evaluated by echocardiography was substantially smaller than that for ECG, particularly at later time points (*n* = 23, *n* = 34, and *n* = 14 for the last three intervals), which likely accounts for the observed variability and precludes any direct statistical comparison between these subgroups. A detailed description of the cardiac abnormalities reported in these patients along follow-up are provided in Supplementary Table 7.

### Long-term serological, parasitological, and molecular follow-up after food-borne Chagas disease: evidence from few reported outbreaks

Long-term serological, parasitological, and molecular follow-up was summarized in Table 7 (Supplementary Table 8). Table 7a showed data on long-term follow-up regarding seronegative conversion and parasite persistence after oral infection were limited. Serological follow-up indicated that anti-*T. cruzi* IgG antibodies may remain detectable for more than 10 years after treatment, as seronegative conversion rates remained low, even after re-treatment. Among patients evaluated at each time point, seronegative conversion was documented in 4.6% after 1 year and 25.3% after 3 years, with a similar proportion observed after 10 years (28.6%). The 60-day regimen of BZL was the most recommended treatment in these outbreaks (10/12), with cases re-treated with BZL due to parasite persistence (27, 28).

**Table 7:** Long-term laboratory follow-up after food-borne Chagas disease: evidence from a limited number of reported outbreaks.

Type of follow-up	Variable	Period evaluated								
		Acute period	<6 months	>6 months <1 year	2 years	3 years	4 years	5 years	>7 years <8 years	>9 years <10 years
**(a)** Serological follow-up	N° of outbreaks	12	7	5	4	2	2	2	1	2
	N° of evaluated patients	232	204	177	186	134	116	128	99	64
	IgG seropositivity (%)	97.5	96.5	78.7	69.3	74.7	93.0	71.9	31.5	56.2
	Seronegativization (%)	0	0	4.6	1.5	25.3	7.0	N.a.	18.6	28.6
**(b)** Parasitological follow-up	N° of outbreaks	10	9	8	8	2	1	1	1	-
	N° of evaluated patients	203	173	177	159	116	106	17	17	-
	Positivity (%)^*^	82.7	6.2	0.12	0.17	0	0	5.9^**^	0	-
**(c)** Molecular follow-up	N° of outbreaks	3	1	1	2	1	1	1	1	2
	N° of evaluated patients	75	3	34	110	98	97	20	82	58
	PCR Positivity (%)^**^	93.0	33.3	29	53	30	32	90	45	65.5

Data are presented according to the time elapsed after completion of antiparasitic treatment. The table summarizes the number of outbreaks, the number of patients evaluated, and the corresponding serological, parasitological, and molecular findings reported at each follow-up interval. Because follow-up duration, laboratory assessment schedules, and the number of patients evaluated varied considerably across outbreaks, the results represent pooled descriptive data and should not be interpreted as longitudinal changes within the same patient cohort. N.a., not available. ^*^All parasitological methods were considered. ^**^The transient increase in parasitological positivity observed at the 5-year follow-up was attributable to a single patient who was also HIV-seropositive, whereas all other evaluated patients remained negative for parasitological tests. No parasitological positivity was detected at the subsequent follow-up evaluation.

Parasitological assessments showed a marked decline in positivity within the first six months after treatment, remaining low up to two years and consistently negative thereafter (Table 7b). However, molecular follow-up was reported in only three outbreaks (3/114, 2.6%). Two of these corresponded to the largest outbreaks described to date (outbreaks #99 and #103), both caused by *T. cruzi* DTU TcI. PCR positivity declined during the first years after treatment, reaching 30–32% by years 3–4. A transient increase was observed at the 5-year evaluation (90%), although this finding was based on a single outbreak involving only 20 patients. PCR positivity subsequently decreased to 45% at 7–8 years and remained detectable in 65.5% (38/58) of patients evaluated after 9–10 years (Table 7c).

## Discussion

Foodborne transmission has been identified as a significant route for emerging infectious diseases, accounting for approximately 15% of all such events worldwide (29). In a broader context, unsafe food is recognized as a major global public health concern, imposing a substantial burden on health systems and economies, including food-borne parasitic diseases (30). Although food-borne *T. cruzi* transmission is increasingly recognized as a public health concern, its epidemiology and clinical impact remain poorly characterized due to fragmented and heterogeneous reporting (31, 32). This systematic review synthesizes nearly six decades of scientific and gray literature to describe the magnitude, geographic distribution, and clinical consequences of food-borne Chagas disease outbreaks, while identifying major evidence gaps.

Acute food-borne Chagas disease outbreaks have been reported across Latin America since the 1960s, with the recent increase likely reflecting improved surveillance, greater recognition of oral transmission, and ecological and social changes, although earlier underdiagnoses cannot be ruled out (31). The outbreaks identified in this review were typically point-source events affecting small- to medium-sized groups, often within family clusters or closed communities sharing contaminated foods or beverages, with attack rates approaching 50%, highlighting the efficiency of the oral route of transmission. Although suspected food vehicles were reported in more than 70% of outbreaks and epidemiological investigations generally supported the proposed source, direct microbiological confirmation of *T. cruzi* contamination was documented in only one outbreak. The median incubation period of 16 days, together with limited food preservation, delayed investigations, and inadequate sampling, likely contributed to recall bias and hindered definitive source attribution. Açaí and sugarcane-derived products were the food sources most frequently implicated in Brazil, whereas a broader range of suspected vehicles -including fruit juices, bush meat, and water- was reported in Colombia and Venezuela, probably reflecting regional dietary habits, food preparation and storage practices, and differences in outbreak investigations. These findings underscore persistent weaknesses in food-borne surveillance and outbreak response, emphasizing the need for improved food safety measures and One Health–based surveillance strategies, particularly for high-risk foods in endemic regions (28, 29).

Demographic data were inconsistently reported, with age and sex available for fewer than two-thirds of outbreaks. Available evidence suggests that oral Chagas disease mainly affects children and young adults, with a slight male predominance, and that mortality occurs across all age groups. Although based on few cases, the high rate of fetal loss among orally infected pregnant women highlights a particularly vulnerable group that remains underreported. The overall case fatality rate was 5.95%, exceeding the 1% reported in a meta-analysis of 2,470 cases evaluated one year after infection (8), but remaining at the lower end of previously reported estimates (5–8%) (1).

Given the 77 deaths and limited cardiological data, severe cardiac complications are likely underestimated, even in treated patients. The scarcity of long-term follow-up further limits assessment of chronic outcomes, while higher fatality rates in some outbreaks may reflect differences in access to care, outbreak size, or parasite factors, precluding causal inference (8).

This systematic review indicates that foodborne Chagas disease outbreaks cluster in tropical and subtropical regions with active sylvatic transmission cycles, and exhibit a seasonal peak during the warmest and rainiest months (33). This pattern overlaps with fruit harvesting, artisanal food processing, and increased triatomine activity in peridomestic environments, likely linking agricultural practices to oral transmission risk (34). Although vector identification was incomplete, *Rhodniu*s and *Panstrongylus* species predominated, and the dominance of commonly considered sylvatic DTUs (especially TcI and TcIV) further supports the association between oral transmission and sylvatic *T. cruz*i cycles. However, the occasional detection of domestic DTUs and mixed infections highlighting the ecological complexity of transmission (35–39).

In addition, oral infection was confirmed using varying combinations of parasitological, serological, and molecular methods, with test-specific positivity rates differing widely due to variations in sensitivity, sampling timing, and laboratory capacity. The absence of standardized diagnostic protocols limits cross-outbreak comparisons, and no studies have yet assessed associations between test positivity and specific *T. cruzi* DTUs.

Prolonged fever was the predominant clinical manifestation, with frequent cardiac abnormalities during the acute phase confirming cardiac involvement as a hallmark of food-borne Chagas disease, often accompanied by headache and facial edema. However, inconsistent clinical reporting likely underestimated disease burden, and limited cardiological follow-up restricted assessment of long-term outcomes. Although electrocardiographic abnormalities tended to decline after treatment, persistent structural cardiac changes were reported in some infected individuals years later, suggesting that long-term cardiac sequelae may occur. The lack of systematic evaluation of digestive involvement remains a major knowledge gap. Parasitological data indicate that early antiparasitic treatment of orally acquired Chagas disease is associated with rapid parasitemia reduction and clinical improvement. However, the genetic diversity of *T. cruzi*, particularly within TcI and its variable susceptibility to BZL, highlights the need for ongoing evaluation of treatment efficacy and alternative or optimized therapies (27, 40, 41). Evidence on long-term serological, parasitological, and molecular outcomes is extremely limited and confined to few outbreaks. Low seronegative conversion rates and occasional late parasite DNA detection suggests heterogeneous treatment responses, but data remain insufficient to define consistent patterns. The lack of integrated long-term molecular and clinical studies further limits understanding of the role of parasite genetic diversity, underscoring the need for standardized longitudinal research to assess long-term outcomes of food-borne Chagas disease (19, 42, 43).

### Strengths and limitations

This systematic review's strengths include a comprehensive search of major databases, supplemented by systematic inclusion of gray literature, and the inclusion of studies in English, Portuguese, and Spanish, enhancing coverage across endemic regions. Overall, this review indicates that foodborne Chagas disease remains an underrecognized and insufficiently characterized public health problem. Quality appraisal revealed marked heterogeneity in reporting, with only 8.8% of outbreaks well documented and over 60% poorly described. The evidence is limited by marked methodological heterogeneity in study design, outbreak investigation, diagnostics, and outcome assessment, which restricts comparability across regions and with vector-borne transmission. Key epidemiological and clinical data were frequently missing or poorly reported, and microbiological confirmation of contaminated food, thorough source investigations, and long-term clinical and laboratory follow-up were rare. Publication bias also cannot be excluded.

Taken together, these methodological and data-related limitations underscore the urgent need to standardize outbreak investigation and reporting practices, strengthen food surveillance in endemic and at-risk settings, and promote well-designed prospective studies to more accurately define the clinical, epidemiological, and public health impact of food-borne *T. cruzi t*ransmission.

### Final remarks

Addressing the oral mode of transmission of Chagas disease requires an integrated, multidisciplinary approach combining clinical care, epidemiological surveillance, and basic research. Despite its documented impact, oral Chagas disease remains underreported in several regions, and outbreak investigations frequently fail to identify contaminated sources or vectors, limiting understanding of transmission dynamics.

By synthesizing data from 114 outbreaks over nearly six decades, this review highlights critical knowledge gaps and the urgent need for standardized, high-quality reporting to improve surveillance, prevention, and clinical management. Strengthening research on oral transmission mechanisms and host responses, together with coordinated action by health agencies, is essential to advance effective control strategies.

## Data Availability

The datasets presented in this study are available in the Supplementary Material and in the IDICER Dataverse repository of the National University of Rosario: https://dataverse.unr.edu.ar/dataverse/idicer.
